# Children’s experiences of a support program during their first year with juvenile idiopathic arthritis — Insights from qualitative interviews

**DOI:** 10.1186/s12969-025-01142-y

**Published:** 2025-09-16

**Authors:** Karina Mördrup, Cecilia Bartholdson, Eva Broström, Johanna Granhagen Jungner

**Affiliations:** 1 Department of Women´s and Children´s Health, Karolinska Institutet, Karolinska vägen 37 A 171 64 Solna, Stockholm, Sweden; 2 Highly specialized pediatric medicine and surgical divisions, Astrid Lindgren Children´s Hospital, Karolinska University Hospital, Stockholm, Sweden

**Keywords:** Children, Arthritis, Juvenile idiopathic, Support-program, Qualitative research

## Abstract

**Background:**

Research reveals that both children and parents often experience fear and anxiety upon being diagnosed with a chronic disease like juvenile idiopathic arthritis. A one-year juvenile arthritis support program (JASP-1) has been developed to offer patient- and family-centered support and education to empower children and their parents in their new situation. However, there is limited knowledge about children's experiences of participating in such support programs. Therefore, this study aimed to describe children’s experiences of participating in JASP-1 during their first year with juvenile idiopathic arthritis.

**Methods:**

Data were collected using individual semi-structured interviews with children who had participated in JASP-1. The interviews were transcribed and analyzed using qualitative content analysis.

**Results:**

Fourteen children between 12 and 17 years of age, with a mean age of 14 years, were interviewed. Three distinct categories were identified. The children reported that their involvement in JASP-1 provided them with a *sense of security through treatment, *a* sense of security through information and support; *and that* contact, visits, and school presence were in balance.*

**Conclusion:**

The results showed that JASP-1 successfully integrated contact through phone calls, visits, and medical and psychosocial support in a satisfactory and accessible way for children recently diagnosed with JIA. This provided them with a comprehensive sense of security. Our findings indicate that patient- and family-centered programs like JASP-1 not only have the potential to enhance and standardize care for children newly diagnosed with JIA but also to contribute to improved and equitable healthcare outcomes in the future.

**Supplementary Information:**

The online version contains supplementary material available at 10.1186/s12969-025-01142-y.

## Background

Juvenile Idiopathic Arthritis (JIA) is one of the most common acquired pediatric rheumatic diseases [1]. It is an illness of unknown etiology that can manifest at any time during childhood. The diagnostic criteria for JIA include joint swelling lasting for more than six weeks, onset before the age of 16, and exclusion of other causes for the joint swelling [1]. JIA encompasses of seven categories, all categorized by inflammatory arthritis [1]. In Nordic countries, the incidence of JIA is approximately 15:100,000 children, with a prevalence of 1:1–2000 [2]. In Sweden around 250 children are diagnosed with JIA annually [2].

Upon receiving information regarding a child’s diagnosis of JIA, previous research indicates that both the child and their family frequently experience heightened levels of anxiety and fear [3, 4]. Additionally, families often find large amounts of information about diagnoses and treatments given at the time of diagnosis difficult to assimilate [3]. A study by van Dijkhuizen [5] found that children and parents require continuous communication and support between their visits to a Pediatric Rheumatology Clinic (PRC) [5].

Few studies are published on how to specifically support children recently diagnosed with JIA. However, those that have been published highlight the need for improved care for children newly diagnosed with JIA through interventions such as patient-centered support and education [6, 7].

It is imperative that the support provided to children and their parents, is tailored to their individual needs and adheres to a patient- and family-centered approach. According to the institute for Patient- and Family-Centered Care, the care is defined as “an approach to the planning, delivery and evaluation of healthcare that is grounded in mutually beneficial partnership among healthcare professionals, patients and families [8]. The use of patient- and family-centered care has the potential to optimize resource allocation and enhance health outcomes [8]. Previous research indicates that patient- and family-centered care positively influences the wellbeing of pediatric patients and their families by fostering greater support and involvement in the care process [9]. Furthermore, a systematic review conducted by Park et al. [10] suggests that patient- and family-centered care is a promising strategy for improving the quality of healthcare delivery [10].

At one of Sweden’s largest pediatric hospitals, a Juvenile Arthritis Support Program (JASP-1) has been developed by a multidisciplinary team of medical doctors (MD), registered nurses (RN), physiotherapists, and one occupational therapist. In addition to the team two research partners with own experiences of JIA were involved in the project to represent patients. The program was developed from May 2017 to March 2018. A pilot study was then conducted, and revision of the program was not considered needed. JASP-1 consists of seven structured patient- and family-centered visits during the first year following diagnosis, including more frequent visits early on. The visits take place week 0, following around week 3, around week 6, week 12, around week 16, week 26 and finally week 52. The duration of the visits typically ranges from approximately 30 min to 1.5 h. At the visits in weeks 0, 12, 26 and 52, the child and the family meet with both the MD and the RN together. After each of these visits, additional time is scheduled with the RN for follow-up questions, support and information. The visit around week 3 is a team visit, where the child, and families are meeting the RN, physiotherapist and occupational therapist one by one. The visits around weeks 6 and 12 are with the RN and can take place either at the clinic or, if preferred by the child and family via phone or a digital meeting. Throughout all visits, a patient- and family-centered approach is consistently employed to establish relationships with families, assess the individual needs of each child and family, provide necessary information, and address any emerging questions or concerns that arise [11]. The program aims to educate and medically and psychosocially support children newly diagnosed with JIA and their parents. Results from a quantitative evaluation study of JASP-1, including 56 eligible children and their parents, showed that the children who completed JASP-1 had better overall assessed health, lower disease impact on daily life, and fewer active joints 12 months after their diagnosis, compared to a control group which did not participate in the program [11].

However, children’s experiences with participating in such support programs are sparsely investigated, and no scientifically published studies have been found to date. Therefore, the aim of this study was to describe children’s experiences of participating in JASP-1.

## Methods

### Study design

This study is part of a large project evaluating JASP-1 in which 56 children and their parents participated. In this study a qualitative design was utilized, adopting an exploratory method to acquire empirical insights and a comprehensive understanding of children’s experiences with JASP-1.

### Participants

Fifteen children aged 8–17 years who had a one year long experience of participating in JASP-1were invited by the first author (KM) to participate in the study using purposive sampling. Purposive sampling is often used in qualitative research for the identification and selection of information-rich participants with experiences about the phenomenon of interest, i.e. it involves selecting participants based on their specific criteria relevant to the research [12].

All 15 children accepted the invitation to participate in individual semi-structured interviews and one child later declined. The interviews were conducted with nine girls and five boys, 12–17 years of age with a mean age of 14, in the period between September 2020 and November 2022 (Table 1). Half of the children were diagnosed with juvenile polyarthritis and the other half with oligoarthritis, enthesitis-related arthritis, psoriatic arthritis, and undifferentiated arthritis.

Inclusion criteria for interviews required that the children participating in this study had been diagnosed with JIA and had completed participation in JASP-1. Additionally, the children needed to be between eight and seventeen years old. Exclusion criteria for interviews included children who did not understand or speak Swedish.

**Table 1:** Demographics for interviewed children

Demographics	*n*		
Age when interviewed	Range 12–17	Mean 14	SD 1,44
Gender *n* (%)			
Female	9 (64)		
Male	5 (36)		

JIA categories	Total *n* = 14	Girls *n* = 9	Boys *n* = 5
Juvenile polyarthritis	7 (50)	5 (36)	2 (14)
Juvenile ankylosing spondylitis	4 (28)	1 (7)	3 (21)
Oligo-arthritis	1 (7)	1 (7)	0 (0)
Juvenile arthritis, unspecified	2 (14)	2 (14)	0 (0)

### Data collection

Data were collected via semi-structured individual interviews using an interview guide that was developed by the first (KM) and second (CB) author. Semi-structured interviews with open questions are, according to Macleud et al. [13], an effective way of enabling children to report information and tell their story. The questions included how the children experienced JASP-1; for example, how they had experienced the intervals between visits, the possibility of contact with the PRC, the information they received and how they received it and what made them feel safe. Follow-up questions such as “Can you explain further?” or “What do you mean?” were frequently asked during the interviews. All interviews were performed by the last author (JGJ) who is an external researcher in this project and thus had no clinical relationship with the children.

Due to the COVID-19 pandemic, the interviews with the children were conducted by phone, after completing JASP-1 which was approximately one year after they received their JIA diagnosis. After conducting 14 interviews with children, no new experiences emerged. Therefore, information power [14], i.e. the more relevant information a sample holds for the study, the fewer participants are needed, because of that no additional participants were recruited. Memos were written directly after each interview. The interviews were, after acceptance from the child and their parents, digitally recorded and transcribed verbatim by the first author (KM) within a couple of weeks after the interviews.

### Data analysis

In this study, the qualitative content analysis process described by Lindgren, Lundman, and Graneheim [15, 16] was used to analyze the transcribed written data. The process of the content analysis was divided into five different phases. When the interviews were completed and transcribed verbatim, the text was read through several times to obtain a sense of the whole context (phase 1). The text was then sorted into meaning units, which are constellations of words or sentences related to the aim of the study (phase 2). The meaning units were then condensed into a description close to the text (phase 3). The first and last authors (KM and JGJ) coded the meaning units and then made a first draft including categorizing the codes into sub-categories which meant collecting codes that were similar in meaning and content (phase 4). Together with all the authors the sub-categories were analyzed and further categorized into three categories (phase 5). Examples of the analysis are shown in supplementary material Table 1. According to Graneheim and Lundman [15], it should be noted that content analysis is not a linear process but a back-and-forth movement between the whole and parts of the text.

## Results

**Fig. 1:**
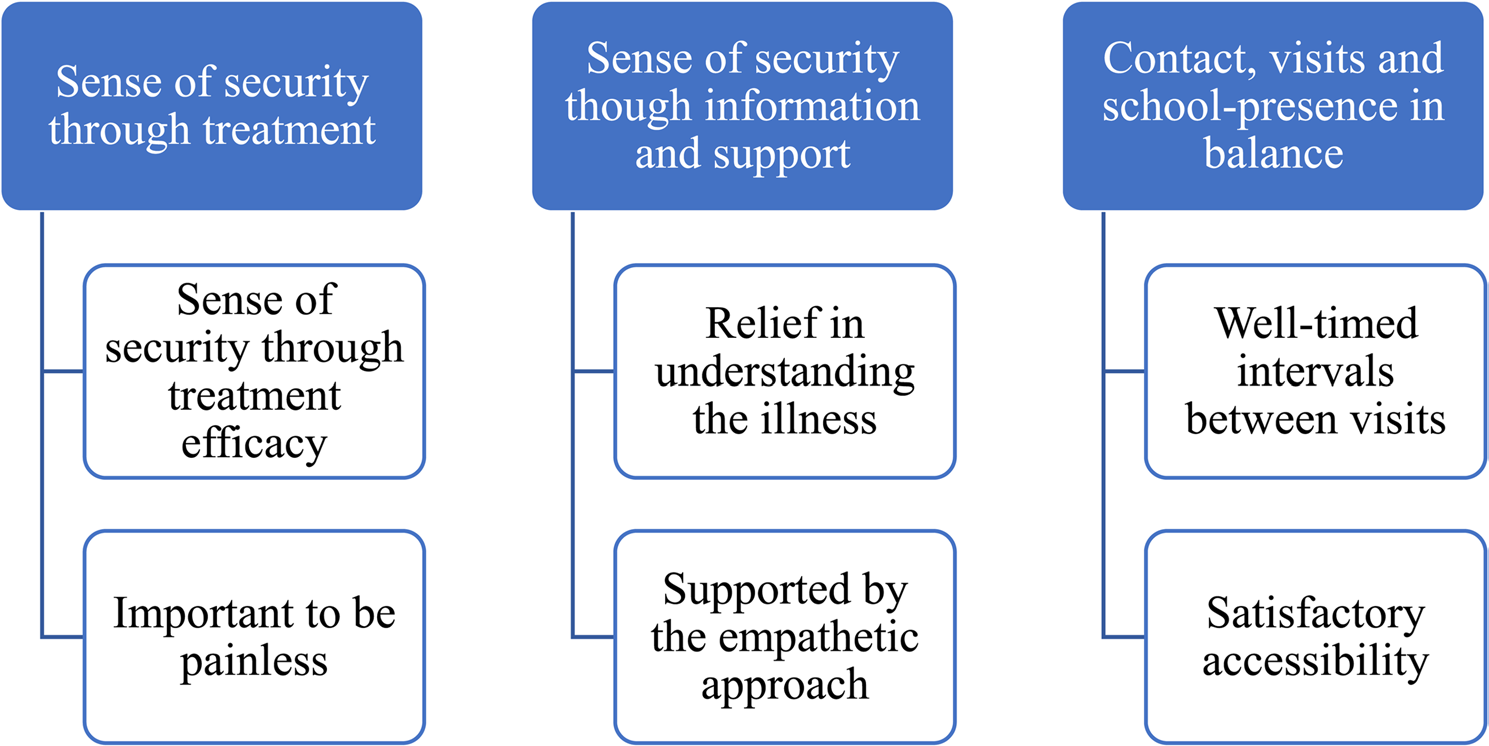
Categories and sub-categories after qualitative content data analysis

Three distinct categories were identified: (1) “sense of security through treatment”, based on the sub-categories “sense of security through treatment efficacy” and “important to be painless”; (2) “sense of security through information and support”, based on the sub-categories “relief in understanding the illness” and “supported by the empathetic approach”; and (3) “contact, visits, and school presence in balance”, based on the sub-categories “well-timed intervals between visits” and “satisfactory accessibility” (Fig. 1).

### Sense of security through treatment

This category is about the participants’ experiences of a sense of security and calm through simply knowing their diagnosis and that JIA treatment was efficient both regarding treating the illness but also in avoiding feeling pain.

#### Sense of security through treatment efficacy

Participants described both initial nervousness about starting their medication as well as happiness and excitement about it. They also expressed a sense of security when receiving treatment that they understood would help them. Moreover, the participants emphasized the importance of being informed about the names of their medications, why and when to administrate them, and understanding the mechanisms through which their treatments operate. The participants reported improvements following joint injections. Furthermore, the participants conveyed that the medication was efficacious, demonstrating a positive impact on their condition and alleviating their pain. This improvement contributed to enhanced well-being in daily life and fostered a sense of security regarding their treatment.

One participant expressed it like this: *I feel safe with my medicines*,* I know that I get well from them*,* and it is safe that I have them to help me (Participant 7*,* 14-year-old girl). *Another participant explained it like this: *I was happy that I was going to get them [the medication red.] and they helped much better and gave more effect*,* so I was excited about it*,* but I was a little nervous (Participant 4*,* 13-year-old girl).*

#### Importance of being painless

The participants expressed how knowledge provided in JASP-1 about pain and pain medication was very important to them and that it made them feel secure knowing they could be painless with medication and that their pain was not dangerous. This was a result of the experience they had prior to their diagnosis with JIA and the start of different treatments. The participants explained how they, prior to the start of treatment, were troubled and concerned about their pain, the swollen joints, and the fatigue. Sometimes the symptoms were so powerful that they went to the emergency room for medical care. Furthermore, they described how they had to develop strategies such as finding new ways of carrying things to reduce pain in the hands and wrists, and that they sometimes just had to sit and watch their friends play or exercise without being able to participate due to symptoms of illness. As a participant expressed it: *most important for me is to take my medication and such so I avoid the pain coming back (Participant 1*,* 14-year-old boy).*

### Sense of security through information and support

This category pertained to the participants’ experiences of receiving information and support, highlighting the empathetic and supportive nature of healthcare professionals (HCP).

#### Relief in understanding the illness

Overall, the participants narrated that it was both a relief as well as a concern and a source of anxiety to receive their JIA diagnosis. Participants expressed overall appreciation for the clear and comprehensible information they received about the illness and its treatment when participating in JASP-1. Furthermore, the participants described how they valued that the information and explanations about the illness and treatment were directed to them, not to their parents, and were given in words they could understand. Furthermore, they acknowledged that receiving information about their illness was done in a structured way that provided a sense of relief, as it explained the cause of their pain, joint swelling, and the possibility they will outgrow the illness. The participants reported feeling supported and assisted by JASP-1 whenever necessary and noted that HCP’s were readily available to provide additional information when required. They also expressed appreciation for the interactions with physiotherapists offered within JASP-1. Additionally, the participants outlined that during JASP-1 they received help in accepting their illness. A participant explained it as follows: *It felt safe that they knew everything (Participant 7*,* 13-year-old girl)*.

Another participant expressed his experiences with JASP-1, saying: *I think they have been good*,* professional*,* and helped me sort of understand. It’s not always easy to get a child to understand their illness*,* but I think they are good at explaining and helping me (Participant 1*,* 14-year-old boy).* Another participant expressed his experiences with the JASP-1 saying, *They were very good at explaining in words that I understood*,* they explained the illness in a very good way*,* and they also explained very well what the medicine did*,* so I understood why I took it and what it did (Participant 10*,* 14-yaer-old boy).*

#### Supported by the empathetic approach

The participants described that the support included HCPs working in JASP-1 as kind and helpful, which made them feel comfortable and looking forward to visiting the PRC. The participants also expressed that they were treated equally and not as sick kids, and they felt that the HCPs genuinely cared about them and were genuinely dedicated to their well-being. Furthermore, the participants felt that the RN in JASP-1 possesses a profound understanding of their emotional state and comprehends the pain they were experiencing, with a reciprocal understanding of the RN’s perspective. Additionally, the participants indicated that they felt understood by the RN in JASP-1 and valued the opportunity of future contact, even during periods of improved health. Moreover, the participants expressed being met with a pleasant and accommodating attitude, providing a sense of calm and helpfulness.

A participant described her experience with JASP-1 like this: *It’s a good and secure place and they are kind to you*,* and you get to know what is happening (Participant 2*,* 12-year-old girl)*. Another described it like this: *She (RN red) truly understands how I really feel*,* and she genuinely understands the pain I am experiencing*,* and I understand her (RN red) (Participant 4*,* 14-year-old girl).*

### Contact, visits, and school presence in balance

This category addresses the participants’ experiences of how the JASP-1 program interacted with their daily life, highlightening a satisfactory balance and how JASP-1 facilitated access to the PRC.

#### Well-timed intervals between visits

The participants expressed that the visits in JASP-1 were fairly spread out and well-planned, with more frequent visits, including closer monitoring, occurring soon after their JIA diagnosis. Additionally, the duration of each visit was deemed appropriate. Moreover, the participants reported that their involvement in JASP-1 caused only minimal disruption to their school attendance. They found that the visits were easily rescheduled when necessary and appreciated the benefits of having a structured plan with scheduled visits through the year.

The participants felt reassured that they were appointed for check-up visits at the PRC and appreciated the continuous contact with the RN in JASP-1 by phone or by a chat connection with the PRC. A participant shared his experience with JASP-1: *Good distance between visits*,* so I didn’t miss anything at school (Participant 10*,* 14-year-old boy)*. Another participant expressed it like this:* They told me what diagnosis I had*,* and yes*,* now the visits are not as frequent*,* I think it feels good*,* now it’s mostly just routine that they check*,* I think it’s nice (Participant 1*,* 14-year-old boy).*

#### Satisfactory accessibility

The participants expressed that the accessibility in JASP-1 has been excellent. The RN was consistently accessible for urgent medical advice or inquiries regarding their illness when needed, which made them feel safe knowing help was just a call or chat away. Furthermore, they valued the telephone communication with the RN in JASP-1, which served to monitor their well-being, address their conditions, and provide feedback as well as the contact with the physio- and occupational therapists.

A participant expressed that: *Everyone has been very good. It feels like you always get through to them*,* and there is always something to talk about” (Participant 5*,* 16-year-old girl).*

## Discussion

This study aimed to describe children’s experiences of participating in JASP-1. This was done by inviting children who had completed JASP-1 to semi-structured individual interviews, where they were given the possibility to tell their story and express their thoughts and feelings. The three distinct categories which were identified during the content analysis were: *sense of security through treatment*; s*ense of security through information and support;* and *contact*,* visits*,* and school presence in balance.*

These categories highlight several key areas; for example, efficacy of treatment and the value of clear information and empathic support, which contribute to the overall sense of security and well-being for children newly diagnosed with JIA.

Our findings in the category *sense of security through treatment* suggest that children exhibited a sense of relief upon discovering different treatment capable of mitigating their illness and associated pain and they highly appreciated that they were involved in the decisions regarding their medications.

As stated in other research, early diagnosis and treatment are essential for mitigating long-term joint damage and improving disease outcomes [17, 18]. Frenkel et al. [17] report an average waiting period of 56 days for PRC appointments. The participants in the present study were reassured by the knowledge that they could access assistance for treatment from the PRC, as outlined in the JASP-1 protocol, whenever necessary. Medication fosters a sense of security in children, with treatment accessibility providing reassurance, while interdisciplinary pain management enhances self-management, functional outcomes, and psychological well-being [19, 20]. We argue that children’s ability to engage in family, social, and educational activities relies on a sense of security, which is fostered through effective treatment and pain management, as also stated by Kotronoulas et al. [21]. This is reinforcing our findings that these interventions are essential for active participation both in and outside of school. Physical exercise is integral to JIA management, alleviating joint pain and stiffness via endogenous opiate stimulation [22]. Delcoigne et al. [23] revealed that children living with JIA face an elevated risk of developing mental health disorders, including sleep disturbances, suicidal behavior, and mood and anxiety conditions due to physiological and/or psychological factors. We argue that this underscores the importance of providing efficient treatment and knowledge through a patient-and family-centered approach to support and help children, together with the family, to develop strategies to manage their illness and pain effectively.

The category *sense of security through information and support* shows that when a child receives a diagnosis such as JIA, they are suddenly in a new, and, for many, vulnerable situation. The present study underlines the critical importance of ensuring that children comprehend their illness and receive empathetic support to foster a sense of security. This aligns with DeCosta et al. [24] findings that newly diagnosed children with chronic illnesses emphasize the need for supportive relationships that provide reassurance without fostering a sense of difference from healthy peers. Based on our results, it is possible to claim that children’s understanding and relationships with HCPs are affected by the patient-and family-centered approach used in JASP-1, and significantly influences the future management of their illness and overall well-being. This is corroborated by Carlson et al. [25] who highlight the importance of patient- and family-centered communication for reducing distress, promoting self-management, and strengthening trust in pediatric care. Furthermore, research indicates that non-medical factors, including family stress and inadequate coping strategies, can impact health-related quality of life [26] and highlight children’s need for treatment management and knowledge when living with JIA [27].

Moreover, our study emphasizes the importance of a patient- and family-centered approach for providing children with clear and comprehensible information about their medical condition, accenting their preference for explanations that are relatable and tailored to their understanding. This is in align with Lin et al. [28], who found that clear, empathetic communication from clinicians fosters respect and security in children. These findings resonate with our research, which underscored the critical role of personalized communication strategies. Specifically, an empathic and patient-and family-centered approach that fosters a calm, friendly, and supportive atmosphere during visits to PRC emerged as essential for enhancing patient experiences and well-being. We argue that consistent care, where the child interacts with the same HCP each time, further contributed to the child’s sense of confidence. Research indicates that children’s healthcare participation benefits from sense of security, continuity of care, and engagement support, while trusting, communicative relationships with HCP further enhance their experiences [25, 29]. This emphasizes the necessity of fostering a supportive and welcoming atmosphere during healthcare interactions.

Moreover, our findings in the category *contact*,* visits and school-presence in balance* indicate that an important factor for the children participating in JASP-1 was that visits to the PRC did not disrupt their school attendance. Prior to being diagnosed with JIA, these children often experienced increased school absence. Haverman et al. [30] and Bouaddi et al. [31] confirm reduced school attendance among children with JIA. This underscores the impact of JIA on school attendance and the importance of minimizing disruptions to education. Children participating in JASP-1 also valued that visits were scheduled more frequently in the beginning of their illness and treatment, when they were feeling more uncertain about their illness and situation. This highlights the importance of providing early, consistent and frequent support to children newly diagnosed with JIA, so they can begin to understand their condition from the very start. In a longer perspective the support can then decrease. This is in line with Heinrich-Rohr et al.’s [32] findings, which emphasize the importance of timely, appropriate healthcare, including frequent initial visits, for effective JIA management and family support [32]. We argue that JASP-1 with the patient- and family-centered approach helps reduce uncertainty and supports better disease management, which can contribute to maintaining school attendance. This is an important aspect due to Pedersen et al. [33] who found that with proper disease management and support, children with JIA achieve academic outcomes comparable to their peers. This further highlights the importance of structured and consistent supportive care to ensure minimal disruption to their education.

The results also revealed that the children’s sense of security was positively influenced by the easy accessibility of the HCP via direct phone or the PRC chat contact. The children expressed that knowing that help was just a call away provided them with a sense of reassurance. These findings align with Ramelet et al. [34], who found that telephone contact with a nurse alleviated morning stiffness and pain in children with JIA.

Interestingly, the qualitative interviews revealed an absence of negative experiences among the children participating in JASP-1. The children consistently reported positive experiences, such as enhanced security through effective treatment, empathetic support, and well-timed intervals between visits that did not disrupt their school attendance. This lack of negative experiences suggests that JASP-1 is highly effective in addressing the needs of children newly diagnosed with JIA, providing them with comprehensive support and information that fosters a sense of security and well-being. Future research could explore whether this positive trend persists across larger and more diverse populations and investigate any potential areas for improvement to ensure the program’s continued success.

### Strengths and study limitations

To increase credibility in this study, the authors have different areas of experience; two of the authors has worked with children with JIA for several years, while others are experienced in interview techniques and/or in qualitative methods. All these areas have contributed to the careful confidence in the findings from the data. All the authors have read and discussed the meaning units and followed the process further to codes, sub-categories, and finally, categories.

To ensure dependability of the findings, the research process has been meticulously detailed, documented, and follows the stages from participant inclusion to interviews, the selection of meaning units, and the development of categories. To aid in transferability, the study setting is facilitated with detailed descriptions and participant demographics.

The qualitative approach provided rich, detailed insights into the experiences and perceptions of children with JIA. Furthermore, the qualitative approach allowed the exploration of various aspects of the children’s experiences, including social, emotional, and medical, which provided a comprehensive understanding of the impact of JASP-1. Another strength of the study is that all participants had the same length of experience with JASP-1, that is, from the time of diagnosis and one year of participation in the JASP-1 program.

A sample size of 14 participants might be a study limitation, since it limits the ability to draw broader conclusions; however, children gave rich narratives and information power was achieved.

As participation in the study was voluntary, there is a risk of selection bias, whereby children who were more confident, interested, or comfortable with the study topic may have been more likely to participate. This could result in the underrepresentation of perspectives from children who were less inclined to volunteer, potentially influencing the diversity and depth of the findings. Consequently, the generalizability of the results may be limited, and this should be considered when interpreting the study’s conclusions.

A further study limitation which has been discussed during the process with JASP-1 was that children who could not speak or understand Swedish were not included in the JASP-1 study and have therefore not participated in the interviews. Also, the experiences of children younger than 12 years of age have not been explored. This can be addressed in future research together with parents’ experiences of participating in JASP-1.

## Conclusion

In conclusion, children have strong beliefs in treatment which emphasize that information and education about treatment is important. JASP-1 seems to effectively integrate visits, contacts, support, and information in an accessible manner for children newly diagnosed with JIA, thereby providing them with an overall sense of security. Our findings suggest that a patient- and family-centered program like JASP-1 has the potential to enhance and standardize care for children newly diagnosed with JIA, contributing to improved and equitable healthcare outcomes in the future.

## Clinical implications

The clinical implications observed during this study include the establishment of closer interactions between the children and the PRC, particularly with the RN. The overall outcomes of the study have thus far been highly positive. Consequently, following the conclusion of the study, the PRC in this study has opted to continue implementing the JASP-1 program. The program has now been expanded to encompass all children diagnosed with JIA, regardless of the timing of their diagnosis in relation to their initial visit. Ensuring that children feel secure regarding their medical condition, treatment, and interactions with HCPs could potentially result in fewer unnecessary visits or visits occurring only when essential. Such developments may, in turn, have a favorable impact on healthcare resource utilization and overall health economics.

## Supplementary Information

Supplementary Material 1.

## Data Availability

No datasets were generated or analysed during the current study.

## Abbreviations

JASP-1: Juvenile Arthritis Support Program for one year
JIA: Juvenile Idiopathic Arthritis
PFCC: Patient- and family-centered care
PRC: Pediatric Rheumatology Clinic
HCP: Healthcare Professionals
MD: Medical Doctor
RN: Registered Nurse
