# Supplementary material for: Children’s experiences of a support program during their first year with juvenile idiopathic arthritis: — Insights from qualitative interviews
Source: Pediatr Rheumatol Online J. 2025 Sep 16;23:92. doi: 10.1186/s12969-025-01142-y (PMC12439369; doi:10.1186/s12969-025-01142-y)
Supplement: Supplementary file 1 — Supplementary Material 1. [file 12969_2025_1142_MOESM1_ESM.docx]

Supplementary material table 1: Phases of data analysis

| Meaning units | Condensed meaning units  Descriptions close to the text | Codes | Sub-categories | Categories |
| --- | --- | --- | --- | --- |
| Good setting and that it is safe, they are kind to you, and you get to know what is happening.  (girl 12 years old) | A safe and good place—they are kind and give info about what will happen | Safe—the staff are calm, kind, helpful and explain well | Supported by the empathetic approach | Sense of security through information and support |
| Pretty good, good distance between visits, so I didn't miss anything at school  (girl 12 years old) | The intervals are pretty good, didn't miss anything at school | The visit intervals were suitably spread out | Well-timed intervals between visits | Contact, visits and school presence in balance |
